# Control of a specific motor program by a small brain area in zebrafish

**DOI:** 10.3389/fncir.2013.00067

**Published:** 2013-04-17

**Authors:** Otto Fajardo, Peixin Zhu, Rainer W. Friedrich

**Affiliations:** Friedrich Miescher Institute for Biomedical ResearchBasel, Switzerland

**Keywords:** zebrafish, optogenetics, motor control, prey capture, J-turn

## Abstract

Complex motor behaviors are thought to be coordinated by networks of brain nuclei that may control different elementary motor programs. Transparent zebrafish larvae offer the opportunity to analyze the functional organization of motor control networks by optical manipulations of neuronal activity during behavior. We examined motor behavior in transgenic larvae expressing channelrhodopsin-2 throughout many neurons in the brain. Wide-field optical stimulation triggered backward and rotating movements caused by the repeated execution of J-turns, a specific motor program that normally occurs during prey capture. Although optically-evoked activity was widespread, behavioral responses were highly coordinated and lateralized. 3-D mapping of behavioral responses to local optical stimuli revealed that J-turns can be triggered specifically in the anterior-ventral optic tectum (avOT) and/or the adjacent pretectum. These results suggest that the execution of J-turns is controlled by a small group of neurons in the midbrain that may act as a command center. The identification of a brain area controlling a defined motor program involved in prey capture is a step toward a comprehensive analysis of neuronal circuits mediating sensorimotor behaviors of zebrafish.

## Introduction

The brain adjusts behavioral actions to events in the environment by transforming sensory input into specific motor output. Even seemingly simple sensory-motor transformations comprise multiple components such as a quantitative evaluation of specific sensory information, sometimes a binary decision, and the computation of appropriate motor commands. A fundamental goal of neuroscience is to understand how each of these tasks are performed by ensembles of neurons, and how these ensembles interact to produce a coherent behavioral response to sensory input (Grillner et al., 2008). An important first step toward this goal is the decomposition of sensory processing and behavioral output into distinct components and the identification of brain areas that control these components. An attractive animal model for systematic analyses of sensory-motor transformations is the larval zebrafish because it is small, transparent, and amenable to sophisticated genetic manipulations (Friedrich et al., 2010). As a consequence, it is possible to activate or silence genetically or spatially defined subsets of neurons by optogenetic approaches and analyze their functions in the context of specific behaviors (Arrenberg et al., 2009; Wyart et al., 2009; del Bene and Wyart, 2012).

Zebrafish larvae have been used to explore the neural basis of simple visual behaviors such as the optokinetic and optomotor responses (Liu and Fetcho, 1999; Neuhauss et al., 1999; Orger et al., 2000, 2008; Orger and Baier, 2005; Huang et al., 2006; Emran et al., 2007; Schoonheim et al., 2010). Visual information is first processed in the retina and then conveyed by retinal ganglion cells to 10 different target areas, the largest of which is the optic tectum (Burrill and Easter, 1994). Optokinetic and optomotor responses are driven by coherent visual motion, which is processed in extra-tectal target areas of retinal ganglion cells (Roeser and Baier, 2003; Gahtan et al., 2005). For the optomotor response, changes in swimming speed and direction were found to be controlled by descending command neurons in the brainstem (Orger et al., 2008). A more complex behavior involving multiple components is prey capture, which emerges ~5 days post fertilization (dpf) when larvae start to feed. This behavior is operant in nature, is induced by small visual stimuli, and consists of a sequence of motor actions that mediate the approach and finally the catch of the prey (Borla et al., 2002; McElligott and O'Malley, 2005; Portugues and Engert, 2009).

Prey capture of zebrafish larvae is strongly reduced in the dark or in blind fish, as measured by the ability to consume Paramecia in a Petri dish (Gahtan et al., 2005; McElligott and O'Malley, 2005). Unlike optokinetic or optomotor reflexes, prey capture is impaired by ablation of the optic tectum (Roeser and Baier, 2003; Gahtan et al., 2005). Tectal output relevant for prey capture may be conveyed to motor nuclei in the hindbrain and spinal cord via the nucleus of the medial longitudinal fascicle (Gahtan et al., 2005) and the reticular formation (Sato et al., 2007). However, the precise set of nuclei involved in prey capture, and the flow of information between these nuclei, has not been established. As prey capture involves a sequence of distinct motor actions, different components of the behavior may be controlled by distinct ensembles of neurons, which could interact serially or in parallel.

Motor behavior leading to prey capture has been decomposed into three phases: orientation toward the prey, approach, and strike (Budick and O'Malley, 2000). During orientation, the fish turns to orient its anterior-posterior body axis toward the prey. This is achieved by a distinctive motor pattern known as J-turn that consists of repetitive unilateral bends of the caudal tail, often accompanied by parallel movements of the pectoral fins (McElligott and O'Malley, 2005). J-turns rotate the body axis of the fish and sometimes result in a slow backward movement (McElligott and O'Malley, 2005). In addition, J-turns are accompanied by convergent eye movements that increase the frontal field of binocular vision, possibly to facilitate stereoscopic estimates of the distance to the prey (Ewert et al., 2001; Bianco et al., 2011). Under laboratory conditions, J-turns including convergent eye movements can be evoked by small moving dots in the frontal visual field (Bianco et al., 2011). These J-turns rotate the fish toward the stimulus, presumably because the stimulus mimics prey. Larger visual stimuli, in contrast, evoke turns in the opposite direction (Bianco et al., 2011). J-turns are therefore a distinct motor program in a sequence of swimming maneuvers during prey capture. However, the brain areas involved in the neural control of J-turns remain unknown. Ablations of the optic tectum in zebrafish larvae reduced small-angle turning movements in the presence of prey (Gahtan et al., 2005), and the tectum contains neurons that are tuned to small moving stimuli (Niell and Smith, 2005; Del Bene et al., 2010). One candidate area that may control J-turns is therefore the optic tectum.

We previously generated transgenic fish expressing channelrhodopsin-2 fused to yellow fluorescent protein (ChR2YFP) under the control of the HuC promoter and the Tet system. Illumination of these larvae with blue light-evoked slow backward movements, a behavior that does not occur spontaneously (Zhu et al., 2009). In this study, we found that this behavior is not caused by uncoordinated muscle movements but by the repeated execution of J-turns. Calcium imaging showed that blue light stimulation evoked widespread neuronal activity in multiple brain regions. However, fiber-optic mapping of behavioral responses revealed that J-turns are specifically triggered by optical stimulation of the anterior-ventral tectum and possibly the underlying pretectum. Focal optical stimulation of this area evoked all characteristics of J-turns in a lateralized fashion. Our experiments therefore identified a small circumscribed brain area that exerts specific control over a defined motor program. These results provide insights into a distinct component of the sensory-motor transformations involved in prey capture.

## Materials and methods

### Animals

Adult fish were maintained at 25°C on a 14/10 h on/off light cycle. HuC:itTA/Ptet:ChR2YFP (lines 2 and 3), Dlx4/6:itTA/Ptet:ChR2YFP (Zhu et al., 2009), and OMP:ChR2YFP (Blumhagen et al., 2011) were outcrossed to wild-type fish (strain AbTÜ/tl) to obtain offspring expressing ChR2YFP and ChR2YFP-negative siblings as controls (“wt”). HuC:itTA/Ptet:ChR2YFP fish were also crossed to nacre(−/−) fish and the F1 generation was incrossed to obtain ChR2YFP-positive fish in the nacre background. Eggs were collected and maintained in E3 medium containing (in mM) 5 NaCl, 0.17 KCl, 0.33 CaCl_2_, and 0.33 MgSO_4_. After sorting at 4–6 dpf, larvae were maintained in standard fish water from the facility and fed powdered fish food. Fish were used between 13 and 25 dpf unless stated otherwise. All experimental protocols were approved by the Veterinary Department of the Canton Basel-Stadt (Switzerland).

### Behavioral analysis of freely swimming larvae

Low-zoom videos from individual zebrafish larvae were collected in a 35 mm Petri dish at a rate of 30 frames per second (fps) using a video tracking system (Zebralab, Viewpoint, France). One light-emitting diode (LED; Luxeon V-Star; 470 nm and 590 nm) equipped with a collimator was placed next to the Petri dish at a distance of ~5 cm and an angle of ~45°. The LED produced ~0.35 mW/mm^2^ at the location of the dish. For experiments testing the effect of light intensity, multiple LEDs (Luxeon Rebel; 470 nm) were arranged around the Petri dish in a circle. Behavioral responses of each larva were tested only once after acclimation of the larvae to the Petri dish for 2–3 min. A trial consisted of three 20 s periods. Blue light was off during the first and the third period and on during the second period. Swimming trajectories were extracted using Viewpoint software and further analyzed in Matlab (Mathworks). Swimming speed was quantified as the mean displacement between adjacent frames, divided by the frame time, and binned into 1 s bins. The “visuomotor on response” was quantified as the mean change in swimming speed during 2 s after light onset, relative to a 10 s baseline period before light onset. The steady state light response was quantified as the mean change in swimming speed during the last 10 s during illumination relative to baseline. The off-response was quantified as the mean change in swimming speed during 20 s after light offset relative to the last 10 s during illumination. Behaviors were visually classified as J-turning when fish moved backwards and the caudal part of the tail was bent repeatedly to one side.

To obtain high-resolution videos, individual zebrafish larvae were placed in a circular arena of 15 mm diameter under a dissecting microscope (SZX 16 with 1xPF Plapo Objective; Olympus) and filmed at a rate of 60 fps using a Grasshopper GRAS-03K2M-C (Point Grey) camera and mot-mot software (Straw and Dickinson, 2009). Fish were illuminated by a blue LED (Luxeon V-Star; 470 nm) placed next to the arena as described above. Blue light was turned on manually when the fish entered the center of the arena. Behaviors were classified by visual inspection of video sequences into one of four categories: “J-turn,” “escape,” “stop,” or “no response.” “J-turn” was scored as described above. “Escape” was defined as an abrupt episode of fast swimming within the first frame after light onset. A more precise analysis of this behavior was not possible because it is too fast to resolve in detail at 60 fps (Liu and Fetcho, 1999). “Stop” was defined as an abrupt cessation of swimming for at least 200 ms after light onset. “No response” was scored when no obvious change in swimming speed and direction were detected relative to a period of ~5 s before light onset.

### Head-fixed behavior

HuC:itTA/Ptet:ChR2YFP larvae used in experiments with head fixation were pre-selected for backward-swimming responses under the conditions described above. Fish were anesthetized in tricaine methanesulfonate (MS-222; 0.1 mg/ml, Sigma-Aldrich) and embedded in 1.5% type VII agarose (low gelling temperature, Sigma-Aldrich) within a 35 mm petri dish. Agarose around the tail and pectoral fins was removed and MS-222 was washed out with fresh fish water to let the fish recover from anesthesia. Wide-field optical stimulation was performed for 3 s with an LED as described above (~0.35 mW/mm^2^). Spatially restricted optical stimulation was performed through the objective of a custom microscope (Euler et al., 2009; Zhu et al., 2012) or using an optical fiber. The microscope was equipped with a 20× objective (Zeiss, NA 1.0), a blue excitation filter (460/50 nm), and a 300 W Xe lamp (LB-LS/30, Sutter Instrument Co.). The mean light intensity in the specimen plane was ~3 mW/mm^2^. However, illumination intensity was not uniform throughout the field of view but substantially higher in the center. For fiber-optic stimulation, optical fibers of 50 or 200 μm diameter (Thorlabs, M14L05 or BFL22-200, respectively) were coupled to a blue laser (CNI; MBL-F-457 nm-500 mW) as described (Zhu et al., 2012). Fibers were held by a hollow metallic rod that was fixed to a rotating mount. The rod was bent by 90° so that the bare end of the fiber was perpendicular to the surface of the fish and could be rotated about the anterior-posterior axis. The rotating mount was held by a motorized manipulator to change fiber position and place the fiber tip close to the fish at each position. The mean light power at the end of the fiber was 110 ± 17 mW/mm^2^ (average over all experiments). To examine the intensity-dependence of behavioral responses, light intensity was varied between 0 and 800 mW/mm^2^. Larvae were filmed at a rate of 60 fps (LED stimulation) or 200 fps (fiber-optic stimulation) using a Grasshoper GRAS-03K2M-C (Point Grey) camera mounted on a dissecting microscope (SZX 16 with 1xPF Plapo Objective; Olympus). In experiments using optical stimulation through an objective, larvae were filmed using the same camera through the condensor of the microscope at 25 fps. Image acquisition was controlled by mot-mot software (Straw and Dickinson, 2009).

Blue light illumination was synchronized to video acquisition by a TTL signal. For coarse behavioral analyses, videos were analyzed visually to classify tail motion as “J-turn,” “forward swimming,” “C-bend,” “escape/struggling,” or “no movement.” “J-turn” was defined as in freely swimming fish as repetitive low-amplitude unilateral bends of the caudal part of the tail (McElligott and O'Malley, 2005). “Forward swimming” was defined as repetitive low amplitude bilateral undulations of the tail (Wyart et al., 2009). “C-bend” was defined as a single large amplitude unilateral bend of the tail, as described previously (Liu and Fetcho, 1999; Sankrithi and O'Malley, 2010). “Escape/struggling” was defined as multiple large-amplitude bilateral bends of the tail (Liu and Fetcho, 1999; Sankrithi and O'Malley, 2010). “No movement” was scored if no tail movements occurred during light stimulation.

For more detailed quantitative analyses of tail movements, videos acquired at 200 fps were analyzed to extract the curvature of the tail in each frame by custom software written in Python. The curvature of the tail was measured using methods similar to those used in a previous study (Bianco et al., 2011). We computed the skeleton of the tail at each frame, divided it in 10 segments, calculated the nine angles between segments and summed over all angles. Resulting traces representing tail bend angle as a function of time were filtered with a low-pass butterworth filter. Anti-clockwise bends are represented by positive angles and clock-wise bends are represented by negative angles. To calculate the asymmetry coefficient from the trace representing tail angle as a function of time, the sign of the absolute tail angle (positive or negative) was determined at each peak of the trace. The asymmetry coefficient was then calculated as the number of peaks with positive sign minus the number of peaks with sign, divided by the total number of peaks. Values close to 0 thus reflect symmetric tail movements while numbers close to one represent highly asymmetric tail movements. Cumulative amplitudes of tail bends were calculated as the sum of the differences between successive peaks and therefore reflect the total amount of tail movement during the behavior. To evaluate eye movements, we fit an ellipse to each eye and extracted the angle of the longer axis relative to the midline of the fish. Resulting traces representing eye angle as a function of time were filtered with a low-pass butterworth filter. Positive changes in angle correspond to eye movements in clockwise direction. Vergence angle was defined as the difference between the angles of the left and right eyes. Rotation toward the midline of either eye results in an increase in vergence angle.

### Confocal imaging

Fish were anesthetized with MS222 (0.1 mg/ml, Sigma-Aldrich) and decapitated. The preparation was glued to the lid of a 35 mm petri dish with tissue adhesive (Vetbond, 3M) and the skin over the brain was removed with a 0.125 mm tungsten dissection probe (WPI). Confocal imaging was then performed using an Olympus Fluoview microscope with a 488 nm excitation laser and an emission filter for eGFP/YFP (505/50 nm).

### Kaede photoconvertion

HuC:kaede transgenic fish (Sato et al., 2006) were anesthetized with MS222 (0.1 mg/ml), embedded in agarose and placed under a dissection microscope (SZX 16 with 1x PF Plapo Objective; Olympus). An optic fiber (50 μm diameter) was coupled to a 405 nm laser (CNI; MDL-III-405nm-250mW) and positioned at the target site using the same procedure as for blue light stimulation. Fish were illuminated for 5 min. Kaede fluorescence was then imaged using a customized two-photon microscope equipped with a 20× water immersion objective (NA 1.0; Zeiss), a Ti:Sapphire laser (SpectraPhysics, Mountain View, CA, USA) at 860 nm, and a photomultiplier-based whole-field detector with emission filters 535/50 nm (green/yellow) and 640/75 nm (red). Data acquisition was controlled by ScanImage and Ephus software (Pologruto et al., 2003; Suter et al., 2010).

### Analysis of melanophores

Fish were anesthetized in MS-222 (0.1 mg/ml, Sigma-Aldrich), embedded in type VII agarose (low gelling temperature, Sigma-Aldrich) and placed under a dissecting microscope (SZX 16 with 1x PF Plapo Objective; Olympus), A picture of the head was taken using a camera attached to the microscope (F-view system; Olympus) while the fish was illuminated from below with an LED lamp integrated into the microscope stand. Images were taken after several minutes of illumination to ensure that melanophores were in the light-adapted state. Using Matlab (Mathworks), pictures were binarized by thresholding, registered by landmarks (snout, eyes, and ears) and summed into a single image. A mask was then drawn over the region over the brain, excluding the eyes and ears. Pixel values represent the number of fish in which the given pixel was covered by a melanophore.

### Calcium imaging

Fish were anesthetized and paralyzed with MS-222 (0.1 mg/ml, Sigma-Aldrich) and Mivacron (0.5 mg/ml, GlaxoSmithKline), respectively, and decapitated. The preparation was glued to a small plastic slide using tissue glue (Vetbond; 3M). Fish water was replaced by cold teleost ACSF (131 mM NaCl, 2 mM KCl, 1.25 mM KH2PO4, 2 mM MgSO4, 10 mM glucose, 2.5 mM CaCl2, and 20 mM NaHCO3) (Mathieson and Maler, 1988) and drugs were washed out. The skin and skull over the brain were removed with a 0.125 mm tungsten dissection probe. The preparation was then incubated at room temperature with rhod-2-AM (6.25 μg in 2 μl DMSO/pluronic F-127 80/20; Invitrogen) for 45 min in ACSF that was continuously bubbled with 95% O2/5% CO2. The preparation was viewed under a custom microscope equipped with a 20× objective (N.A. 1.0; Zeiss) (Euler et al., 2009; Zhu et al., 2012). An optic fiber (200 μm diameter) coupled to a blue laser was placed close to the preparation at an angle of ~30° so that the light beam was directed approximately to the anterior tectum on one side of the brain. Conventional epifluorescence imaging was performed using a Xe arc lamp (Sutter Instrument Co. LB-LS/30, 300 W), a 545/25 excitation filter, a 605/70 emission filter, and a CCD camera (CoolSnap; Photometrics). Data was acquired using custom software written in Igor (Wavemetrics) and analyzed using custom software written in Python. Fluorescence signals were expressed as relative changes in fluorescence intensity (ΔF/F) in each pixel with respect to a pre-stimulus baseline.

### Statistical analysis

All summary data are presented as mean ± s.e.m unless noted otherwise. Age of fish in days is reported with the standard deviation (SD). Statistical tests used are stated in the Results.

## Results

### Optical stimulation of motor behavior in zebrafish larvae expressing channelrhodopsin-2

**Figure 1:**
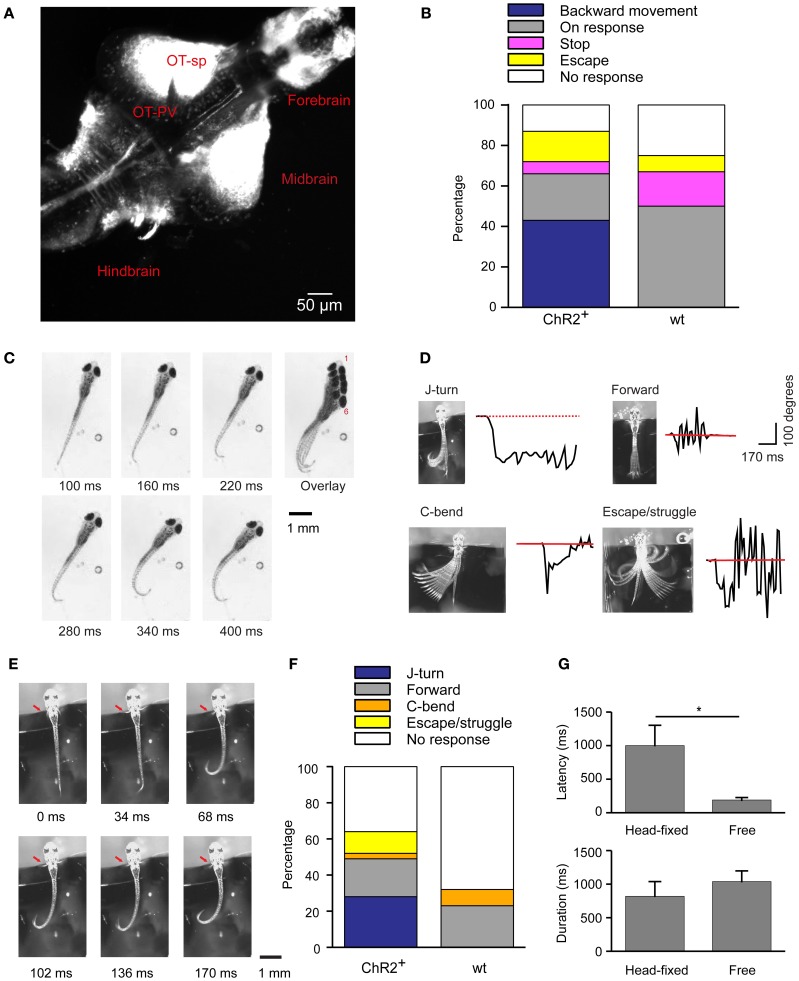
**Optical stimulation evokes J-turn in HuC:itTA/Ptet:ChR2YFP zebrafish larvae. (A)** Pattern of ChR2YFP expression in the brain of a HuC:itTA/Ptet:ChR2YFP larva (17 dpf; z-projection of a confocal stack). OT-sp, Optic tectum-superficial layers; OT-PV, Optic tectum-periventricular layer. **(B)** Classification of behavioral responses to blue light stimulation in freely swimming zebrafish larvae. Backward movement was the dominant response in HuC:itTA/Ptet:ChR2YFP transgenics (ChR2^+^; *n* = 47 fish; one trial per fish) but never occurred in wt siblings (wt; *n* = 12). **(C)** Video sequence of a freely swimming HuC:itTA/Ptet:ChR2YFP larvae during an episode of backward movement. Note unilateral bends of the caudal tail, a characteristic of J-turns. Overlay illustrates net backward movement and rotation. **(D)** Examples of four different behaviors observed in head-fixed fish. Black traces show the curvature of the tail as a function of time. Red line represents the resting angle (straight tail). **(E)** Video sequence of a J-turn response in a head-fixed larva. Note unilateral bends of the caudal tail and symmetric movement of the pectoral fins (red arrow). **(F)** Classification of behavioral responses to blue light stimulation in head-fixed larvae. J-turns were frequently observed in HuC:itTA/Ptet:ChR2YFP (95 trials in 17 fish) but never in wt siblings (44 trials in 8 fish). **(G)** Latency (time from blue light onset to the initiation of motor response) and duration of J-turns evoked by blue light stimulation in freely swimming (*n* = 12) and head-fixed (*n* = 15) HuC:itTA/Ptet:ChR2YFP larvae (mean ± s.e.m.). ^*^p = 0.019, Student's *t*-test.

In a previous study, multiple lines of HuC:itTA/Ptet:ChR2YFP transgenic zebrafish were created that express two transgenes: (1) the Tet activator, itTA, under the control of the HuC promoter, and (2) channelrhodopsin-2 (ChR2) fused to yellow fluorescent protein (YFP; Chr2YFP) under the control of Ptet, a Tet responder element (Zhu et al., 2009). As a result, Chr2YFP is expressed in many, though not all, neurons at larval stages (Figure 1A). In some of these lines, exposure to blue light triggered slow backward movement of larvae. However, because motor behavior was analyzed at low resolution, it remained unclear whether backward movements were generated by a coordinated motor program, or whether they were caused by uncoordinated muscle contractions (Zhu et al., 2009).

To address this question we illuminated freely swimming zebrafish larvae between 13 and 24 dpf (mean ± SD: 16 ± 3 dpf) with a blue LED for 20 s and monitored motor behavior by video imaging at high magnification. The light intensity at the specimen was ~0.35 mW/mm^2^. Under baseline conditions, larvae showed normal swimming behavior, consisting of bouts separated by periods of little or no movement. Light onset triggered different motor behaviors that were classified as “backward movement,” “visuomotor on response,” “stop,” “escape,” and “no response.” “Backward movement consisted of a slow backward displacement, often associated with lateral excursions or rotations. This behavior was never observed spontaneously in clean water. The “visuomotor on response” is a well-described transient increase in mean swimming speed after a change in ambient light levels, mainly due to an increase in bout frequency, that is mediated by the visual system (Burgess and Granato, 2007). “Escape” was defined as a sudden episode of high-speed swimming after light onset. “Stop” was defined as a cessation of swimming, and “no response” was scored when no obvious change in swimming behavior was observed.

Among HuC:itTA/Ptet:ChR2YFP larvae (*n* = 47; one trial per fish), 43% responded to blue light stimulation with backward movement, 23% showed a visuomotor on response, 6% stopped swimming, 15% escaped, and 13% showed no response. In wt siblings that did not express ChR2 (*n* = 12), backward movement was never observed but 50% showed visuomotor on responses, 17% stopped swimming, 8% escaped, and 25% showed no response (Figure 1B). In addition, we selected three HuC:itTA/Ptet:ChR2YFP larvae that swam backwards during blue light illumination and tested their response to illumination with an amber LED (590 nm, ~0.35 mW/mm^2^). All of these fish showed a strong visuomotor on response to amber light but no backward motion. These results confirm that backward movement is triggered by activation of ChR2. The observed frequency of backward movement was somewhat lower than in a previous study (43% vs. ~80%) (Zhu et al., 2009), possibly because the expression levels of ChR2YFP decreased slightly over successive generations.

High-magnification videos showed that fish performed slow, repeated, unilateral tail bends during backward movement episodes. These bends were usually limited to the caudal part of the tail while the proximal trunk appeared stiff (Figure 1C). This motor behavior resulted in a net backward motion and often also rotated the fish (Figure 1C, **overlay**). Within a trial, tail bends were usually exclusively to one side (45% to the right, 50% to the left; 5% both sides). This behavior closely resembles J-turns, a motor pattern displayed during prey capture (McElligott and O'Malley, 2005; Bianco et al., 2011). The latency (time from blue light onset to the beginning of tail movement) and the duration of this behavior were 186 ± 50 ms and 1038 ± 50 ms, respectively.

To characterize the motor behavior underlying optically-evoked backward movement in more detail we immobilized larvae by embedding the head in agarose and filmed motor behavior at 60 Hz. The tail and pectoral fins were free [age of fish: 17.5 ± 4 dpf (mean ± SD); range: 13–24 dpf]. Tail movements evoked by a 3 s illumination with a blue LED (~0.35 mW/mm^2^) were classified into motor patterns that have been associated with different swimming patterns: J-turning, forward swimming, C-bends, escape/struggling, and no movement (Figure 1D). J-turning was defined as repeated unilateral bends of the caudal tail. Forward swimming was defined as continuous and symmetric undulations of the entire tail with intermediate amplitude and frequency. C-bends were defined as single, fast unilateral bends of the tail. This well-characterized motor program orients the fish away from an aversive stimulus during an escape response (Liu and Fetcho, 1999). Escape swimming/struggling was defined as high-amplitude, bilateral tail movements more vigorous than forward swimming. No response was scored when no obvious movements were observed. Head-fixed HuC:itTA/Ptet:ChR2YFP fish responded to light with J-turns (Figure 1E; Movie S1), although with lower probability than under free-swimming conditions (*n* = 95 trials in 17 fish, Figure 1F), indicating that head-fixation increased behavioral thresholds. Wt siblings never performed J-turns and usually showed no response (*n* = 44 trials in 8 fish, Figure 1F). The latency of optically-evoked J-turns in HuC:itTA/Ptet:ChR2YFP fish (1001 ± 302 ms; *n* = 15 fish) was longer than in freely swimming animals (Student's *t*-test, *p* = 0.019) but the duration was similar (819 ± 200 ms, *n* = 15 fish; Student's *t*-test, *p* > 0.05, Figure 1G).

**Figure 2:**
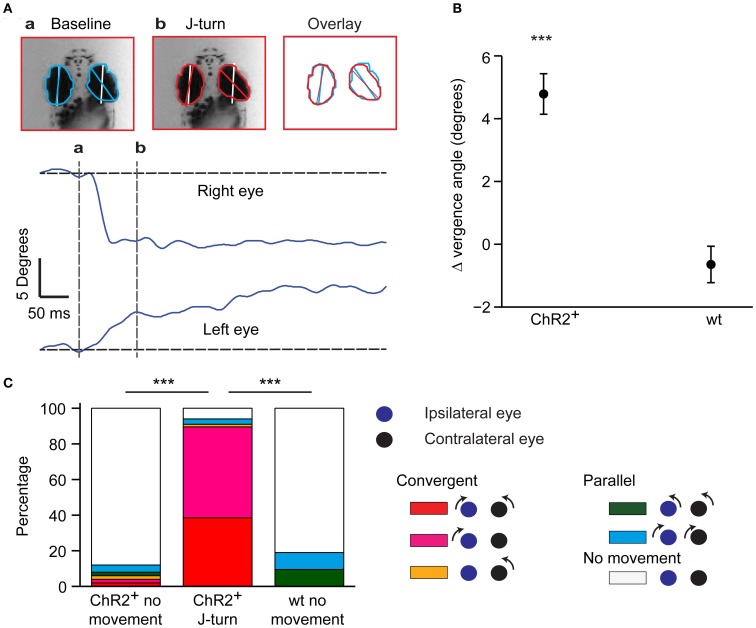
**Convergent eye movements during optically-evoked J-turns. (A)** Eye position before (a) and during (b) a J-turn evoked by optical stimulation in a HuC:itTA/Ptet:ChR2YFP larva. White lines show orientation of anterior-posterior axis; colored lines show angle of the eye. Overlay shows convergence of eyes during J-turn. Traces show angular changes in eye position as a function of time. **(B)** Mean change in eye vergence angle (±s.e.m.) evoked by blue light stimulation in HuC:itTA/Ptet:ChR2YFP larvae (ChR2^+^) and wt siblings. Positive change indicates convergence of eyes. ^***^p < 0.001, Student's *t*-test. **(C)** Classification of eye movements in HuC:itTA/Ptet:ChR2YFP larvae that did not respond to blue light (*n* = 49), in HuC:itTA/Ptet:ChR2YFP larvae responding with J-turns (*n* = 65), and in wt siblings, which never responded with J-turns (*n* = 32). An eye movement was defined as an angular change in eye position >2°. Convergent eye movements were closely associated with J-turns. ^***^p < 0.001, Chi-square test for comparison of the frequency of convergent eye movements.

In zebrafish hunting real or virtual prey, J-turns often involve simultaneous forward and backward swings of both pectoral fins (“in-phase fin movements”). During forward swimming, in contrast, pectoral fins are moved in anti-phase or held still (Thorsen et al., 2004). Moreover, J-turns are often associated with convergent eye movements (Borla et al., 2002; McElligott and O'Malley, 2005; Bianco et al., 2011). We found that unilateral tail movements evoked by optical stimulation of head-fixed HuC:itTA/Ptet:ChR2YFP larvae were accompanied by in-phase movements of both pectoral fins in almost all trials (95%) (Figure 1E, **arrow**; Movie S1). During behavior classified as forward swimming, in-phase movements of the pectoral fins occurred in only 10% of trials while anti-phase movements were frequently observed. Moreover, optical stimulation that evoked J-turning often also evoked convergent eye movements (Figure 2A; Movie S1). To quantify eye movements we measured the angular difference in the orientation of the two eyes (vergence angle) before and during exposure to blue light. This difference was clearly positive in HuC:itTA/Ptet:ChR2YFP larvae, indicating convergence of the eyes, but near 0 in wt siblings [150 trials in ChR2 positive fish and 44 trials in WT fish, Student's *t*-test, *p* < 0.001; age of fish: 18 ± 3.4 dpf (mean ± SD), range: 13–22 dpf; Figure 2B]. In HuC:itTA/Ptet:ChR2YFP fish, convergent eye movements occurred in the majority of trials that evoked unilateral tail movements (*n* = 65) but were virtually absent when optical stimulation failed to evoke tail movements (*n* = 49). No convergent eye movements were observed in wt fish (*n* = 32; Figure 2C). Hence, optical stimulation evoked a coordinated motor program with all characteristics of J-turns. We therefore refer to the motor behavior during optically-evoked backward movements as J-turning.

During prey capture, J-turns orient the fish toward the prey before the strike (McElligott and O'Malley, 2005). J-turns do therefore not normally occur in isolation but are embedded in a sequence of other motor programs (McElligott and O'Malley, 2005). Moreover, episodes of J-turning are brief, which explains why J-turning is not always associated with a net backward movement during prey capture. Optogenetic stimulation of HuC:itTA/Ptet:ChR2YFP larvae therefore triggered the repeated execution of an isolated motor pattern.

### Further characterization of optically-evoked J-turns

**Figure 3:**
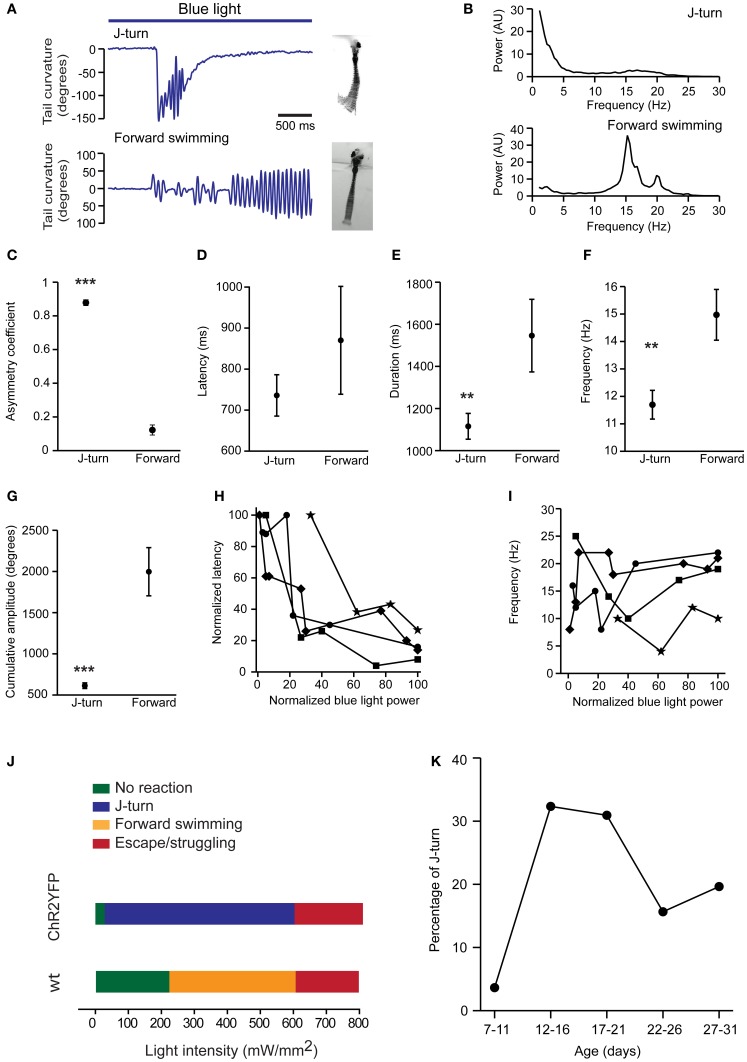
**Quantitative comparison of J-turns and forward swimming. (A)** Examples of tail curvature as a function of time during episodes of forward swimming and J-turning. Fish were stimulated with blue light starting at the onset of the trace. **(B)** Power spectral analysis of tail curvature during forward swimming and J-turns (average over all trials). **(C–G)** Asymmetry of tail movements, latency of motor response, duration of motor behavior, mean frequency of tail beats, and cumulative amplitude of tail beats for optically-evoked J-turns (*n* = 138 trials in 12 larvae; mean ± s.e.m.) and forward swims (*n* = 35 trials in 9 larvae). Mean frequency and cumulative amplitude were calculated over the total duration of the behavior, including short periods of inactivity. ^**^p < 0.01; ^***^p < 0.001; Student's *t*-test. **(H,I)** Latency and frequency of J-turns as a function of light intensity in four HuC:itTA/Ptet:ChR2YFP larvae. Because behavioral thresholds varied between individuals, data for each larvae were normalized to the maximum intensity used for each larva. Latencies were normalized to the maximum latency observed in each larva. **(J)** Dependence of behavioral response on intensity of blue light stimulation. Colored bars indicate the most frequently observed response as a function of light intensity in HuC:itTA/Ptet:ChR2YFP larvae (*n* = 10 fish) and wt siblings (*n* = 8). **(K)** Probability of J-turn responses as a function of age.

To further quantify motor behavior in head-fixed fish we selectively illuminated the midbrain with a laser through an optic fiber (diameter, 200 or 50 μm; light intensity 110 ± 17 mW/mm^2^), filmed motor behavior at 200 Hz, and quantified the curvature of the tail [18 ± 3.6 dpf (mean ± SD), range: 13–24 dpf]. We restricted our analysis to J-turns and forward swimming because C-bends and escape/struggling swimming were too fast to track even at 200 Hz. Forward swimming was characterized by bilateral low amplitude tail bends, while J-turns were characterized by repetitive unilateral bends of the caudal tail (Figure 3A). To quantify the frequency of tail bends in these two behaviors we analyzed tail curvature as a function of time (Figure 3A), computed power spectra of each trial, and averaged power spectra over trials. Power spectra of J-turns had a peak at low frequency (<5 Hz) reflecting the unilateral excursion of the tail, and another, broad peak between 15 and 20 Hz that reflects repetitive bending of the caudal tail. This frequency is similar to the tail bend frequency during naturally occurring J-turns or slightly lower (McElligott and O'Malley, 2005; Bianco et al., 2011). Small differences between optically-evoked and naturally occurring behaviors may be due to head fixation or differences in age. Power spectra of forward swims showed one or multiple peaks between 15 and 20 Hz (Figure 3B).

Asymmetry of tail movements to the left and right sides was quantified by a coefficient that varies between 0 (completely symmetric tail movements) and 1 (tail bent to only one side). This analysis confirmed that tail movements were highly asymmetric during J-turns but nearly symmetric during forward swims (J-turns: 0.88 ± 0.01, *n* = 138 trials in 12 fish; forward swims: 0.12 ± 0.02, *n* = 35 trials in 9 fish; *p* < 0.001, Student's *t*-test; Figures 3A,C). The latency of J-turns and forward swims was not significantly different (J-turns: 736 ± 50 ms; forward swimming: 870 ± 131 ms; *p* > 0.05, Student's *t*-test; Figure 3D). The duration of the behavior was determined as the period during light stimulation when obvious tail movements occurred. During this period, fish sometimes showed multiple episodes of tail movements, separated by short episodes of low activity. On average, the duration of J-turn behavior (1115 ± 61 ms) was significantly lower than the duration of forward swims (1542 ± 172 ms; *p* = 0.004, Student's *t*-test, Figure 3E). Nevertheless, the mean duration of J-turn behavior appeared substantially longer than episodes of J-turning during prey capture (Borla et al., 2002; McElligott and O'Malley, 2005; Bianco et al., 2011). J-turns usually ceased before the end of the 3 s light stimulation. This observation is consistent with transient J-turn responses to stimulation with virtual prey (Bianco et al., 2011) and may reflect fatigue, inactivation of ChR2, or adaptation. The mean frequency of tail beats, averaged over the duration of the behavior, was significantly lower during J-turning than during forward swimming (12 ± 0.5 vs. 15 ± 0.9 Hz; *p* = 0.005, Student's *t*-test, Figure 3F). The cumulative amplitude of tail beats (sum of absolute amplitude peaks) was also significantly lower during J-turns (613 ± 34° vs. 1997 ± 293°; *p* < 0.001, Student's *t*-test, Figure 3G), consistent with the lower duration and tail beat frequency. Increasing the intensity of the light stimulus dramatically reduced the latency of J-turn responses but did not affect tail beat frequency (Figures 3H,I). These results further support the conclusion that optical stimulation triggered the execution of a stereotyped motor program.

Using optical stimulation with a 200 μm fiber, the intensity threshold for evoking J-turn in HuC:itTA/Ptet:ChR2YFP larvae was 32 ± 8.5 mW/mm^2^ (mean threshold in 10 larvae). For light intensities up to ~600 mW/mm^2^, J-turns were the behavioral response that was most frequently observed (Figure 3I). Beyond ~600 mW/mm^2^, J-turn responses were mixed with escape or struggling-like responses, presumably because the light stimulus produced noxious heat. In wt fish, no behavioral response was observed for light intensities below 217 ± 16 mW/mm^2^ (mean threshold in 8 larvae). Higher intensities sometimes produced forward swimming, which may reflect a visuomotor response to the light. Above ~600 mW/mm^2^, light stimulation occasionally produced escape or struggle responses, as observed in HuC:itTA/Ptet:ChR2YFP larvae (Figure 3J). These results confirm that J-turning is a distinct motor program that is evoked by optical stimulation of neurons and overrides other motor behaviors.

We next examined the age-dependence of J-turn responses in freely swimming HuC:itTA/Ptet:ChR2YFP fish stimulated with a blue LED (~0.35 mW/mm^2^). The probability of triggering J-turns increased abruptly after 11 dpf and decreased somewhat after 21 dpf (Figure 3K). The reason for this age-dependence remains unclear. A possible reason for the relatively late onset is that the motor programs mediating J-turns or the upstream command centers are not fully developed before 11 dpf. However, J-turns occur during prey capture already at 5 dpf and can be evoked by virtual prey at the same age (Budick and O'Malley, 2000; Bianco et al., 2011). Alternatively, optical stimulation may be inefficient at early stages because neuronal response thresholds are higher. The decline in the probability of J-turning after 21 dpf could be due to decreased penetration of the light into the brain because skin and bones become less transparent. Alternatively, fish may use motor programs others than J-turns for prey capture at later stages. Moreover, it is possible that expression of ChR2YFP in neurons involved in J-turns is downregulated at later developmental stages. However, no obvious change in expression pattern was observed around 21 dpf (see below), although expression eventually becomes restricted to defined types of neurons in adult fish (Zhu et al., 2009).

**Figure 4:**
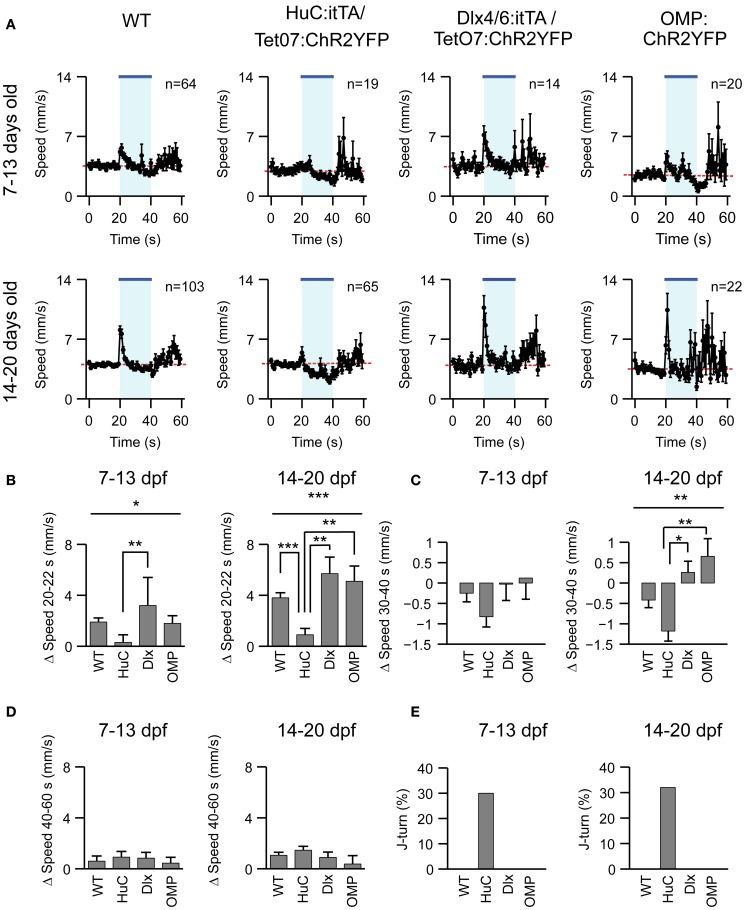
**Effects of optical stimulation in different transgenic fish lines. (A)** Swimming speed as a function of time (1 s bins) in four different zebrafish lines and two different age groups before, during and after a 20 s exposure to blue light (bar; LED). *n*, number of fish. **(B–D)** Mean change in swimming speed during a 2 s period after light onset (20–22 s), a 10 s period before light offset (30–40 s) and a 20 s period after light offset (40–60 s). **(E)** Percentage of J-turn responses evoked by blue light stimulation in different fish lines. Error bars show s.e.m. ^*^p < 0.05; ^**^p < 0.01; ^***^p < 0.001; ANOVA and Bonferroni *post-hoc* test.

To explore whether J-turns are triggered by the activation of specific subsets of neurons we first examined the effect of blue light exposure on freely swimming larvae of two other ChR2-expressing lines, Dlx4/6:itTA/Ptet:ChR2YFP (Zhu et al., 2009) and OMP:ChR2YFP (Blumhagen et al., 2011). Dlx4/6:itTA/Ptet:ChR2YFP transgenic fish express ChR2 in a large number of GABAergic interneurons (Zerucha et al., 2000; Zhu et al., 2009), while OMP:ChR2YFP transgenics express ChR2 in a subset of olfactory sensory neurons (Sato et al., 2005). Larvae of these lines, as well as HuC:itTA/Ptet:ChR2YFP and wt larvae, were exposed to blue light for 20 s while swimming speed was measured continuously (Figure 4A). Changes in swimming speed relative to a baseline, measured over 10 s before light onset, were then quantified in three time windows: 0–2 s after light onset, 10–20 s after light onset, and 0–20 s after light offset. The first time window (0–2 s after onset) captures the visuomotor on response, a well-described transient increase in swimming speed evoked by a sudden change in ambient light levels (Easter and Nicola, 1996; Burgess and Granato, 2007). The second time window (10–20 s after onset) was chosen to quantify steady-state effects of blue light, while the third time window (0–20 s after offset) was chosen to quantify after-effects of light exposure (“off-response”). Results were averaged in two age groups (7–13 and 14–20 dpf).

A clear visuomotor on response after light onset was observed in all lines except HuC:itTA/Ptet:ChR2YFP (Figure 4B). Moreover, HuC:itTA/Ptet:ChR2YFP, but not other lines, showed pronounced negative changes in swimming speed during the steady-state light response (Figure 4C). These effects were observed as a trend in young larvae (7–13 dpf) and statistically significant for the second age group (14–20 dpf). No significant differences were observed between off-responses of different lines in any age group (Figure 4D). Previous observations indicated that an obvious visuomotor on response is rare in HuC:itTA/Ptet:ChR2YFP larvae because they swim backwards instead (Zhu et al., 2009). Consistent with this conclusion, J-turns were observed in ~30% of the trials with HuC:itTA/Ptet:ChR2YFP but never in larvae of other lines (Figure 4E). These results confirm that J-turns are not evoked by non-specific optical stimulation of neurons or by visual input. Rather, the optical stimulation of J-turns appears to require a particular pattern of ChR2 expression, suggesting that J-turns were triggered by the stimulation of specific neurons.

### Optical stimulation evoked broadly distributed neuronal activity

To examine the distribution of activity in the brain evoked by optical stimulation in HuC:itTA/Ptet:ChR2YFP larvae [19 ± 3.2 dpf (mean ± SD), range: 14–23 dpf] we exposed the brain from the dorsal side in an *ex-vivo* preparation of the head and loaded neurons with the calcium-sensitive dye rhod-2-AM by bath incubation. Light pulses from a blue laser (duration, 500 ms) were directed at the midbrain through an optical fiber (diameter, 200 μm) from one side. Calcium signals were measured by wide-field epifluorescence imaging with a CCD camera. Because optical stimulation interfered with quantitative fluorescence imaging, changes in indicator fluorescence were measured immediately after the optical stimulus.

**Figure 5:**
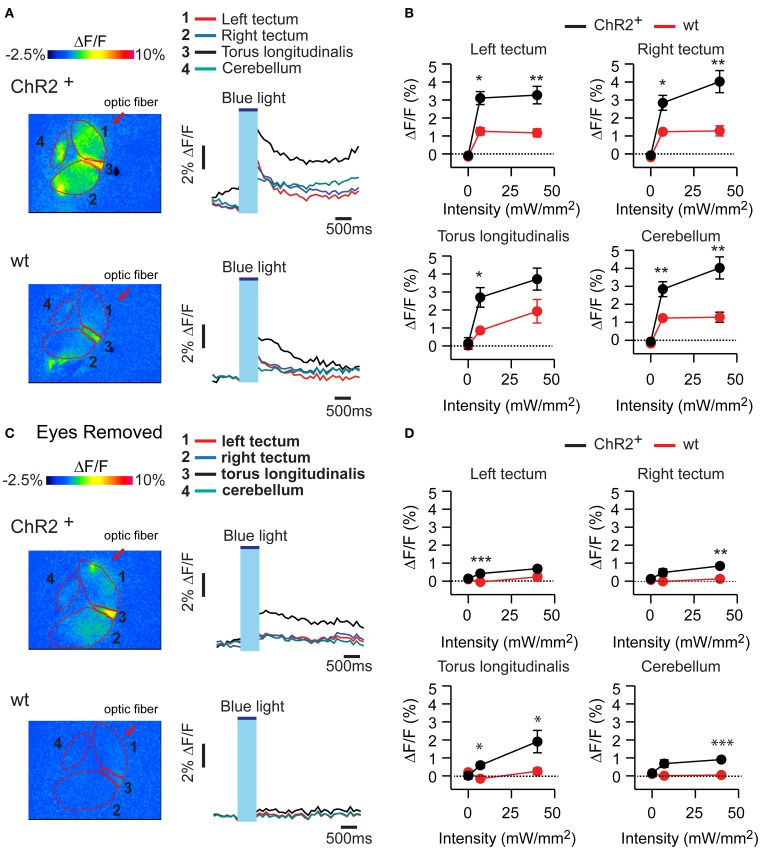
**Calcium signals evoked by optical stimulation. (A)** Changes in fluorescence intensity of the calcium indicator rhod-2 in an *ex-vivo* preparation of the larval head after optical stimulation with blue light. Top: HuC:itTA/Ptet:ChR2YFP; bottom: wt. Four different brain areas are outlined (left optic tectum, right optic tectum, torus longitudinalis, cerebellum). **(B)** Mean changes in fluorescence intensity in these brain areas evoked by stimuli of two different intensities. Error bars show s.e.m. **(C,D)** Same experiments performed in preparations without eyes. Error bars show s.e.m. ^*^p < 0.05; ^**^p < 0.01; ^***^p < 0.001; Student's *t*-test.

In HuC:itTA/Ptet:ChR2YFP fish, optical stimulation at two different intensities (7 mW/mm^2^ and 40 mW/mm^2^) evoked calcium signals in various brain areas including the tectum, torus longitudinalis, and cerebellum. This response is expected to contain different components evoked by activation of ChR2 and by visual stimulation through the eyes. Consistent with this assumption, optical stimulation of wt siblings evoked smaller calcium signals (Figures 5A,B) that were almost completely abolished after surgical removal of the eyes (Figures 5C,D). In HuC:itTA/Ptet:ChR2YFP fish, in contrast, a substantial signal remained after removal of the eyes, confirming that optical stimulation can directly stimulate neurons in the brain by activation of ChR2. This residual response was widespread, which could be due to the broad expression of ChR2, the propagation of locally evoked activity, or both. The distribution of light-evoked activity does therefore not provide specific information about the brain areas controlling J-turns.

### J-turning depends on light intensity

**Figure 6:**
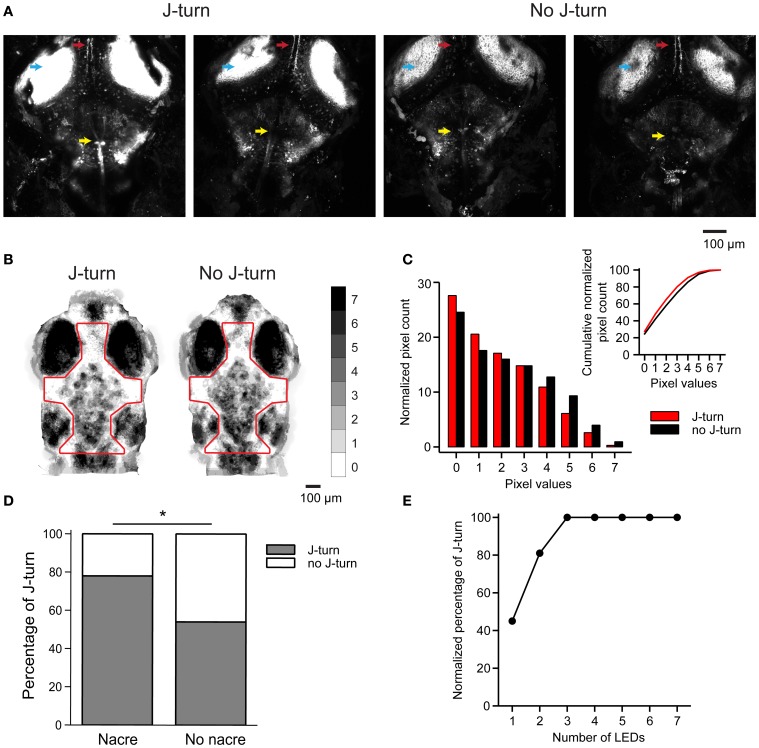
**J-turn responses depend on efficiency of optical stimulation. (A)** ChR2YFP expression in two HuC:itTA/Ptet:ChR2YFP larvae that responded to blue light stimulation with J-turns (left) and two siblings that did not respond (right). Arrowheads depict the optic tectum, the tecto-toral pathway, and a prominent group of hindbrain neurons. Images are z-projections of confocal stacks acquired using the same settings. **(B)** Sum of binarized and registered images of seven HuC:itTA/Ptet:ChR2YFP larvae that responded to blue light stimulation with J-turns and seven siblings that did not respond. Gray levels indicate the number of larvae in which each pixel was covered by pigment. Pigmentation was then quantified within the outlined area. **(C)** Distribution of pixel counts in the images in **(B)**. The distribution is shifted to the left for images from larvae that responded with J-turns, indicating less pigmentation (Kolmogorov-Smirnov test, *p* < 0.001). Insert shows cumulative distributions of pixel counts. **(D)** Probability of J-turning in HUC:itTA/Ptet:ChR2YFP fish in the nacre background and in pigmented siblings. ^*^p = 0.021, Chi-square test. **(E)** Normalized probability of J-turn responses in pigmented HuC:itTA/Ptet:ChR2YFP larvae (*n* = 20) as a function of light intensity (number of LEDs).

To explore which brain areas are involved in optically-evoked J-turns we first correlated the occurrence of J-turns to the expression pattern of ChR2YFP. Although the HuC promoter can drive expression in most or all neurons, expression of ChR2YFP in HuC:itTA/Ptet:ChR2YFP fish was not pan-neuronal because it was controlled by the Tet system, which often restricts expression to specific subsets of the neurons that are normally targeted by the promoter driving itTA expression. This “sparsening” appears to depend on the integration site of one or both of the transgenes (HuC:itTA and Ptet:ChR2YFP) and therefore results in expression differences between founder lines (Zhu et al., 2009). Individual larvae from more than one founder were exposed to three blue light stimulations (LED, 3 s; ~0.35 mW/mm^2^). Eight larvae were then selected that responded with J-turn to all three stimulations, as well as four larvae that did not respond to light [19 ± 3.2 dpf (mean ± SD), range: 15–22 dpf]. In each of these larvae, ChR2YFP expression was analyzed by confocal microscopy. As expected, the expression of ChR2YFP showed distinct differences between individuals in some brain areas (Figure 6A). For example, the tecto-toral pathway (Figure 6A, red arrow) or a prominent group of reticular neurons (Figure 6A, yellow arrow) expressed ChR2YFP in some fish but not in others. However, none of the observed expression differences correlated with the occurrence of J-turns (Figure 6A). We noticed, however, that the expression of ChR2YFP was stronger in larvae that performed J-turns, particularly in the optic tectum (Figure 6A, ligh blue arrow). These results suggest that J-turns are initiated by neurons that express Chr2YFP in most or all founder lines such as the optic tectum, and that other factors, such as expression levels, are responsible for the behavioral variations between individuals.

The observed correlation between expression levels and J-turn responses raises the possibility that some fish failed to respond to optical stimulation because the effective stimulation intensity was too low. In live fish, the effective stimulation intensity is expected to depend on pigmentation because access of blue light to the brain is blocked by melanophores. The pattern of melanophores differs between individuals but is usually more similar between siblings than between larvae from different lines (Engeszer et al., 2008). We therefore analyzed melanophore patterns in seven HuC:itTA/Ptet:ChR2YFP larvae that responded to blue light LED stimulation with J-turn and compared them to melanophore patterns of seven HuC:itTA/Ptet:ChR2YFP fish that failed to respond [18 ± 4.8 dpf (mean ± SD), range: 13–25 dpf]. Images of the head were binarized by thresholding to extract melanophores, registered by landmarks (snout, eyes, and ears), and summed for each group (Figure 6B). The value of each pixel therefore represents the number of fish in which the corresponding location was covered by a melanophore. We found no consistent difference in the spatial pattern of melanophores between the two groups. However, the mean coverage of the head by melanophores, as evaluated from the distributions of pixel values, was significantly higher in fish that did not respond to optical stimulation (*p* < 0.001, Komogorov-Smirnov test; Figure 6C). It is therefore possible that individual fish failed to respond to light stimulation because melanophores reduced the effective stimulus intenstity.

To test whether a difference in the effective optical stimulation intensity can account for inter-individual variations in behavioral responses we outcrossed HuC:itTA and Ptet:ChR2YFP to the nacre mutant, which lacks melanophores. Because nacre is recessive, siblings with and without melanophores could then be obtained from the same crossings. The probability of J-turn responses to light stimulation was significantly higher in fish with the nacre phenotype than in pigmented siblings (78% of 41 nacre fish vs. 54% of 62 wt siblings; *p* = 0.021, Chi-square test, age = 15 dpf; Figure 6D). Moreover, when light intensity was increased by additional LEDs the probability of J-turning increased until it reached a saturating level (*n* = 20 fish, age = 14 dpf, Figure 6E). These results indicate that inter-individual variations in J-turn responses were, at least in part, due to differences in the effectiveness of light stimulation.

### Spatial mapping of brain areas controlling J-turns

**Figure 7:**
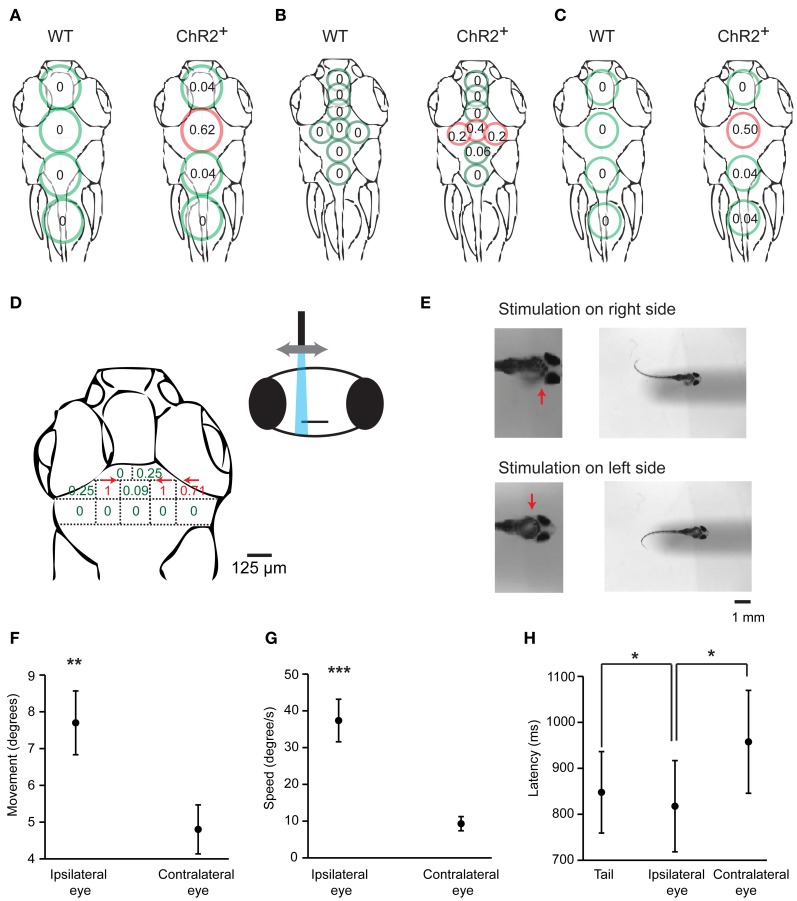
**Mapping of J-turn responses by local optical stimulation. (A)** Approximate regions illuminated with blue light from an epifluorescence lamp (circles) superimposed on an outline of the zebrafish head (dorsal view). Numbers within circles show the probability of J-turn responses to optical stimulation in each location in HuC:itTA/Ptet:ChR2YFP larvae (ChR2^+^; *n* = 8 fish, 3 trials at each position) and wt siblings (*n* = 5 fish, 3 trials at each position). **(B)** Same with more restricted blue light illumination (field aperture closed; *n* = 5 fish, 3 trials at each position for ChR2^+^ and wt siblings). **(C)** Mapping of J-turn responses to blue light stimulation through a vertical optic fiber (diameter, 200 μm; *n* = 8 fish for ChR2^+^ and *n* = 7 fish for wt; 3 trials at each position). **(D)** Mapping of J-turn responses to optical stimulation in the midbrain using a vertical optical fiber with 50 μm diameter. Numbers indicate the probability of J-turn responses at the corresponding positions (*n* = 4 fish, between 3 and 13 trials each position). Arrows indicate the direction of tail bends when the probability of J-turning was >0.25. **(E)** Images showing tail bends evoked by optical stimulation in the anterior tectum with a small optical fiber (50 μm) on different sides. Arrows show the side of the brain that was stimulated. **(F,G)** Mean angular movement and movement speed of the ipsilateral and contralateral eyes evoked by stimulation with an optical fiber (50 μm) above the anterior tectum on one side. **(H)** Mean latency of eye movements and tail movements. All error bars show s.e.m. ^*^p < 0.05; ^**^p < 0.01; ^***^p < 0.001; Student's *t*-test.

To identify brain areas involved in J-turning we mapped behavioral responses of head-fixed HuC:itTA/Ptet:ChR2YFP larvae to focal optical stimulation at different sites. Coarse mapping was performed by illuminating circular areas ~400 μm in diameter using a microscope equipped with a 20× objective, a blue excitation filter (460/50 nm), and an epifluorescence lamp (LB-LS/30, 300W, Sutter Instrument Co.). Tail movements were filmed through the condenser. We illuminated four regions in the midline over the forebrain, midbrain, hindbrain, and rostral spinal cord and found the probability of evoking J-turns to be maximal in the midbrain [*p* = 0.62, 24 trials in 8 fish, 19 ± 2.9 dpf (mean ± SD), range: 16–23 dpf; Figure 7A]. Optical stimulation in all other regions, as well as broad illumination of the tail (not shown), evoked little or no J-turning. Finer mapping with smaller light stimuli (~150 μm in diameter) in the same fish confirmed that J-turn responses were triggered selectively in the midbrain (*p* = 0.4 in a central area; 15 trials in 5 fish at each position, Figure 7B). Wt siblings showed no response to illumination of any region [15 trials in 5 fish at each position, 19 ± 3.1 dpf (mean ± SD), range: 16–23 dpf; Figures 7A,B).

To map behavioral responses more precisely we used a blue laser coupled to an optical fiber. This method produces slightly divergent cones of light with relatively sharp edges whose diameter depends on the diameter of the optical fiber (Arrenberg et al., 2009; Zhu et al., 2012). Using a relatively large fiber (200 μm diameter) oriented perpendicularly to the brain surface and an average light intensity of 110 ± 17 mW/mm^2^, J-turns could again be evoked in the midbrain but not in adjacent brain regions of HuC:itTA/Ptet:ChR2YFP larvae (*p* = 0.5 in the midbrain; 24 trials in 8 fish at each position; age: 18 ± 3.4 dpf (mean ± SD), range: 13–21 dpf] while wt fish did not respond [21 trials in 7 fish at each position; age: 18 ± 3.6 dpf (mean ± SD), range: 13–21 dpf; Figure 7C]. Finer mapping with a 50 μm fiber perpendicular to the dorsal brain surface revealed two regions in the anterior midbrain where J-turns could be triggered in each trial. These regions were ~65 μm lateral to the midline and bilaterally symmetric. As the fiber was moved away from these regions, the probability of evoking J-turns decreased sharply [*n* = 8 fish; multiple trials at each position; age: 17 ± 4 dpf (mean ± SD), range: 13–22 dpf; Figure 7D]. J-turns can therefore be triggered specifically by stimulation of a small, circumscribed region in the anterior midbrain.

We further observed that the side of stimulation determined the direction of tail movements: illumination on the right side evoked tail bends exclusively to the left and vice versa (*n* = 26 trials with 200 μm fiber; *n* = 35 trials with 50 μm fiber; Figure 7E). Furthermore, when optical stimulation on one side evoked convergent eye movements (*n* = 25 trials in 4 fish), the magnitude and speed of eye movements were significantly larger on the ipsilateral side (paired student *t*-test, *p* < 0.001, Figures 7F,G). The latency of the ipsilateral eye movement was significantly shorter than the response latency of the contralateral eye (paired student *t*-test, *p* = 0.047, Figure 7H). Moreover, the response latency of the ipsilateral eye was significantly shorter than the latency of the tail movement (paired student *t*-test, *p* = 0.02, Figure 7H) whereas the latency of the contralateral eye movement was significantly longer. Convergent eye movements always included the ipsilateral eye, but not always the contralateral eye (Figure 2C). Eye movement is therefore asymmetric and, on average, initiated before tail movement, consistent with results obtained by visual stimulation with virtual prey (Bianco et al., 2011). These results show that J-turn behavior is strongly lateralized.

### Localization of a brain area that controls J-turns

**Figure 8:**
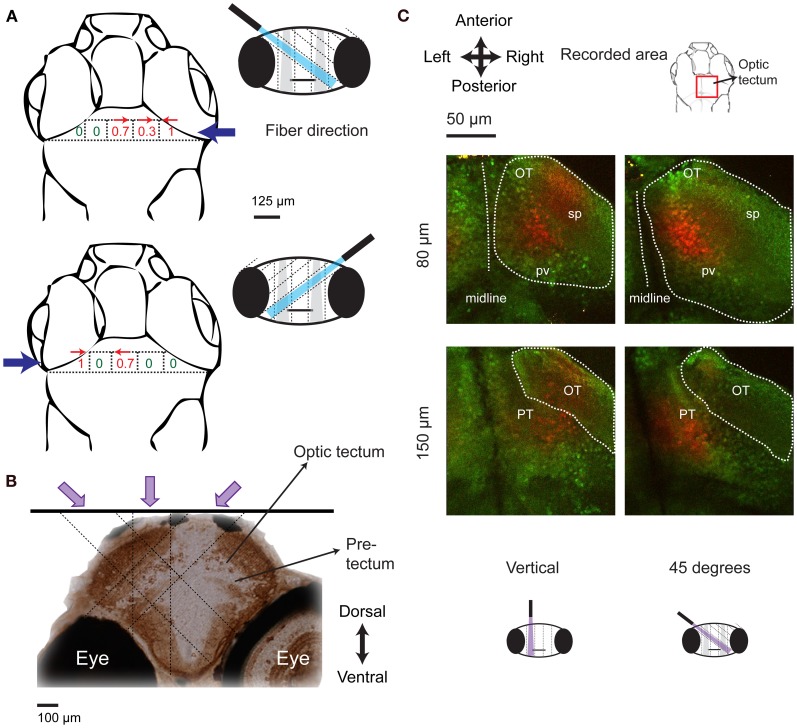
**Localization of the brain area triggering J-turns in 3-D. (A)** Probabilities of evoking J-turns in the midbrain of HuC:itTA/Ptet: ChR2YFP larvae with a 50 μm optical fiber tilted by 45° to the left or to the right. Numbers indicate the probability of J-turn responses at the corresponding positions (*n* = 3 fish, 3 trials at each position). Arrows indicate the direction of evoked tail bends when the probability of J-turning was >0.25. **(B)** Schematic illustration of the localization of the brain area triggering J-turns by triangulation. Image shows a coronal section of the zebrafish brain stained with anti-GFP, approximately at the rostro-caudal position where J-turns were evoked with highest probability (level of anterior tectum). Dashed lines indicate the light paths of optical stimuli (vertical and 45°) that produced J-turns with maximal probability. Light paths intersect in a region containing the anterior-ventral optic tectum (avOT) and part of the pretectum. **(C)** Photoconversion of kaede after illumination with UV light through an optic fiber (50 μm) oriented vertically or at 45°. The fiber was positioned at sites that produced maximal probabilities of J-turn. Images (z-projections of small multiphoton stacks) are shown at two depths for two fish illuminated at different angles. Most photoconverted neurons (red) were found in the avOT. OT, optic tectum; pv, periventricular layer; sp, superficial layers; PT, pretectum.

Mapping with a vertical fiber identified the x–y coordinates of a brain area triggering J-turns but cannot identify its location in the third dimension because the light is not focused. We therefore rotated the 50 μm optic fiber by 45° and re-mapped tail movements evoked by optical stimulation at different positions in the anterior midbrain. When the fiber came in from the left, tail movements in a specific direction were evoked at distinct sites (Figure 8A, **top**) that were displaced leftwards compared to the locations where equivalent tail movements were evoked by vertical fiber stimulation (Figure 7D). Likewise, stimulation sites producing equivalent tail movements were displaced rightwards when the fiber came in from right (Figure 8A, **bottom**). The depth of the region activated during J-turn can be thus be estimated by triangulation (see schematic illustration in Figure 8B). This procedure indicates that the brain region triggering J-turns is situated in the anterior tectum or the underying pretectum, approximately 80–150 μm below the brain surface.

To anatomically localize the brain area activated by optical stimulation we traced the stimulation light in fish expressing the photoswitchable protein kaede under the control of the HuC promoter (HuC:kaede) (Sato et al., 2006; Arrenberg et al., 2009). When illuminated with violet light, fluorescence emission of kaede changes from green to red (Sato et al., 2006). A 50 μm optical fiber oriented vertically or at 45° was placed at the positions where the probability of evoking J-turns by blue light was maximal in HuC:itTA/Ptet:ChR2YFP fish. Fish were then illuminated through the fiber with light from a violet laser (405 nm) for 5 min. Red and green fluorescence of Kaede was examined in a volume around the stimulation site by multiphoton microscopy. Photoconverted kaede protein was detected in columns of ~50 μm diameter along the projected paths of the violet light (*n* = 8 in 4 fish for vertical illumination and *n* = 3 in 3 fish for 45°, age: 15–16 dpf; Figure 8C). Columns produced by stimulation at different angles (vertical or 45°) overlapped mainly in the ventral part of the anterior optic tectum. This area includes fibers coming from the retina and cell bodies in the periventricular layer of the tectum (Meek and Schellart, 1978; Scott and Baier, 2009; Robles et al., 2011). Some cells in a structure ventral to the optic tectum, presumably the pretectum, were also photoconverted (Figure 8C). The identity of this area could not be determined unequivocally because precise anatomical atlases at the developmental stages examined here are not available. Comparisons with anatomical data from earlier stages suggest that this area may be located between structures identified as the pretectum and the early migrated pretectum (M1) (Mueller and Wullimann, 2002), or coincide with terminal field AF-9, which may differentiate into the nucleus pretectalis pars dorsalis and/or pars ventralis during development (Burrill and Easter, 1994). These results demonstrate that J-turns were triggered by stimulating the anterior-ventral optic tectum (avOT) and/or an adjacent pretectal nucleus.

## Discussion

Optogenetic mapping of behavioral responses in transgenic zebrafish expressing ChR2 revealed that the activation of avOT or the adjacent pretectum evokes J-turns, a well-defined motor program involved in prey capture. J-turns consisted of highly coordinated motor components and could not be evoked elsewhere in the brain, suggesting that the anterior-ventral tectum acts as a command center controlling a defined motor program. These findings provide insights into senorimotor transformations underlying a complex behavior that is critical for the survival of zebrafish larvae.

### Mapping of a defined motor behavior in zebrafish

A previous study reported that larval HuC:itTA/Ptet:ChR2YFP zebrafish respond to wide-field optical stimulation with slow backward movements and rotations (Zhu et al., 2009). High-resolution video analysis in freely swimming and head-fixed larvae revealed that this behavior is not due to uncoordinated muscle contractions but produced by the repeated execution of J-turns. J-turns must involve the coordinated control of multiple muscle groups in different body parts to achieve unilateral caudal tail bends, parallel movements of pectoral fins, and convergent eye movements. Quantitative behavioral analyses showed that these motor components co-varied in different trials, exhibited a high degree of temporal coherence, and were strongly lateralized. Optical stimulation did therefore not activate different muscle groups independently but triggered the execution of a coordinated motor program, indicating that optical stimulation activated a command center upstream of local motor control.

Optical stimulation evoked calcium signals throughout multiple brain areas, consistent with the widespread expression of ChR2. These results indicate that optical stimulation was not restricted to specific subsets of neurons but activated multiple types of neurons throughout the brain. Motor output, however, was distinct, highly coordinated and reproducible. The most likely explanation for this unexpected finding is that the motor program for J-turns is activated at low threshold and suppresses other behavioral reactions. Consistent with this assumption, HuC:itTA/Ptet:ChR2YFP larvae exposed to amber (590 nm) instead of blue light produced visuomotor on responses, the typical visual response of wt larvae, rather than J-turns. Likewise, blue light stimulation under head-fixed conditions triggered J-turns in HuC:itTA/Ptet:ChR2YFP larvae but forward swimming in wt larvae throughout a broad intensity range. Hence, optically-evoked J-turns appear to override visuomotor on responses. These results suggest that different motor programs are controlled in a competitive fashion such that the execution of one motor program suppresses the execution of others. Well-defined, stereotyped motor programs may therefore be controlled by different command centers that inhibit each other (Ewert et al., 2001; Humphries et al., 2007; Orger et al., 2008). Obviously, such a competitive and modular organization of motor control can establish coherent behavioral outputs by avoiding interference between conflicting motor programs.

Observations in zebrafish and many other species suggest that neuronal circuits controlling stereotyped motor programs are, at least in some cases, spatially localized in the brain (Ewert, 1967; Schaefer, 1970; Syka and Radil-Weiss, 1971; Stein and Clamann, 1981; McHaffie and Stein, 1982; Al-Akel et al., 1986; Salas et al., 1997; Herrero et al., 1998; Valentine et al., 2002; Gahtan et al., 2005; Saitoh et al., 2007; Schoonheim et al., 2010; Miri et al., 2011). Consistent with this notion, we found that J-turns can be triggered efficiently and specifically by optical stimulation within a small volume (<100 μm in diameter) containing avOT and a small part of an adjacent pretectal area. This volume was localized with high precision in three dimensions by optical stimulation with a thin fiber at different angles. The volume is relatively deep and contained neurons that expressed ChR2YFP at similar levels as neurons in other tectal areas. It is therefore unlikely that behavioral responses were triggered specifically in this area because optical stimulation was more efficient than in other regions. Rather, our results suggest that this area is functionally specialized to control J-turns.

Experiments using electrical stimulation in different vertebrate species indicate that the optic tectum or its homolog, the superior colliculus, is involved in orienting movements of the eyes, pinnae, head, and body toward a target (Ewert, 1967; Schaefer, 1970; Syka and Radil-Weiss, 1971; Stein and Clamann, 1981; McHaffie and Stein, 1982; du Lac and Knudsen, 1990; Herrero et al., 1998; Valentine et al., 2002; Saitoh et al., 2007). As J-turns orient zebrafish toward small, prey-like objects, our results are consistent with this general notion. Moreover, we found that optical stimulation in avOT also produced coordinated eye movements, which may be another motor behavior involved in orientation (Bianco et al., 2011). The precise mechanisms by which tectal circuits convert sensory inputs into motor commands are, however, poorly understood. The zebrafish may therefore provide an excellent experimental model to study these sensorimotor transformations. Experiments in various species also demonstrated that electrical stimulation in other brain areas such as the brainstem can produce coordinated motor output, presumably by activation of command neurons (Sterman and Fairchild, 1966; Grillner and Shik, 1973; Grillner et al., 2008). As the brainstem contains various nuclei that transmit information to the spinal cord, it is likely to further process motor signals before relaying them to central pattern generators in the spinal cord (Grillner et al., 2008). The functions and interactions of brain stem nuclei are, however, not well-understood. As shown by this study and others (Arrenberg et al., 2009; Scott and Baier, 2009; Schoonheim et al., 2010), optogenetic mapping in zebrafish is a promising approach to address these questions.

### Tectal control of motor output

The volume where optical stimulation evoked J-turns coincided mostly with avOT. This tectal subregion encompasses ~15% of the total tectal volume and is not delineated from other parts of the tectum by obvious anatomical boundaries. Since photoconversion of kaede was observed also in an adjacent pretectal area it cannot be excluded that J-turns were elicited by stimulation of this pretectal area, or by simultaneous stimulation of the pretectum and avOT. However, the number of photoconverted neurons in the pretectum was low. Moreover, optical stimulation at angles and positions that should have favored stimulation of the pretectal area did not efficiently trigger J-turns. We therefore conclude that J-turns were probably evoked by stimulation of avOT.

J-turns can be evoked by small moving objects (“virtual prey”) in a subregion of the visual field (Bianco et al., 2011). One possibility is that avOT receives retinal input from this subregion. If so, the optical stimulation used in our experiments may have evoked J-turns by creating a “neural illusion” of a small moving object in the appropriate visual subfield. Such an illusion could be evoked if optical stimulation produced specific responses of the corresponding retinal axons. However, this is highly unlikely because optical stimuli were stationary and J-turns could be evoked by wide-field illumination with blue light. J-turns were therefore likely triggered by stimulation of tectal neurons. Unlike retinal afferents, many tectal neurons responded more efficiently to small moving bars than to large moving bars (Del Bene et al., 2010). A “neural illusion” of a small moving object may therefore be created by direct stimulation of these neurons.

Wide-field stimulation would be expected to create conflicting “neural illusions” simultaneously in many virtual positions in the visual field. Moreover, neurons responding to small bars are not restricted to avOT but distributed throughout the optic tectum (Niell and Smith, 2005; Del Bene et al., 2010). Activation of tectal neurons tuned to small objects can therefore not explain the observed J-turn responses without additional assumptions. One possibility is that avOT contains a larger number or a higher density of neurons responding to small objects than other tectal regions. Widespread optical stimulation may therefore evoke strong activity in avOT, which may trigger J-turns and suppress other behavioral responses. Alternatively, avOT may be functionally different from other tectal areas and specialized for the control of J-turns. One possible specialization of avOT could be projections to specific motor nuclei. It may therefore be hypothesized that the tectal output to motor areas controlling J-turns arises specifically in avOT. Targets in more lateral or caudal directions should therefore not evoke J-turns but possibly trigger other motor behaviors. Consistent with this hypothesis, J-turns during prey capture correlate with activity in a tectal area consistent with avOT, as revealed by calcium imaging in freely swimming larvae (Muto et al., 2013).

The finding that J-turns could be evoked specifically in avOT but not in other tectal areas raises the possibility that the tectum is not a homogeneous structure but contains functionally specialized subregions. Consistent with this hypothesis, electrical stimulation in different regions of the tectum elicited different motor reactions in goldfish (Salas et al., 1997; Herrero et al., 1998). As input from retinal ganglion cells to the optic tectum appears to be topographic and continuous (Xiao et al., 2005; Gosse et al., 2008), functional specializations of tectal subregions may be established by region-specific neuronal circuits within the tectum, or by specific projections from tectal subregions to other brain areas. This hypothesis could be tested experimentally by tracing tectal outputs from avOT and other tectal subregions to downstream brain areas such as the reticular formation (Sato et al., 2007). A spatial patterning of tectal outputs would appear useful if stimuli in different regions of the visual field convey information that is relevant for the control of different behaviors. Alternatively, output from the tectum may be homogeneous, and different behavioral responses to activity in specific tectal areas may be generated by downstream target areas that analyze tectal output. Various results indicate that the optical tectum is not a purely sensory area for processing of visual information but also involved in other functions including motor control (Meek and Schellart, 1978; Goodale, 1996; Gandhi and Katnani, 2011). This notion is reinforced by the finding that specific optical stimulation of the avOT produces a distinct motor output.

Previous studies demonstrated that ablation of the optic tectum impairs prey capture in zebrafish (Gahtan et al., 2005) and goldfish (Springer et al., 1977). This impairment was associated with a reduction in small-angle turns, possibly J-turns (Gahtan et al., 2005). AvOT may therefore be both sufficient and necessary to evoke J-turns, although this hypothesis remains to be tested by additional experiments. Even if avOT is a hub for the control of J-turns, other brain areas are also likely to be involved in this behavior. For example, the work by Ewert and colleagues in toads indicates that visual stimuli signaling prey and predators stimulate neurons in the optic tectum and in the pretectum, respectively, that interact when both types of stimuli are presented simultaneously (Ewert et al., 2001). Prey capture by zebrafish involves a sequence of behavioral components that need to be coordinated by interactions between specific groups of neurons, presumably across brain areas. The identification of avOT as an area that can trigger J-turns is a step toward the functional understanding of this network.

### Conflict of interest statement

The authors declare that the research was conducted in the absence of any commercial or financial relationships that could be construed as a potential conflict of interest.

## Supplementary material

Movie S1 **Light-evoked J-turns and convergent eye movements in a head-fixed HuC:itTA/Ptet:ChR2YFP fish.** The optic tectum was illuminated with blue light through an optical fiber (200 μm diameter) for the duration of the movie. Light onset was at the first frame. The original frame rate was 200 Hz; the movie is slowed down by a factor of four.
